# Supplementary figures and images for: Role of Pink1-mediated mitophagy in adenomyosis
Source: PeerJ. 2023 Nov 30;11:e16497. doi: 10.7717/peerj.16497 (PMC10693823; doi:10.7717/peerj.16497)

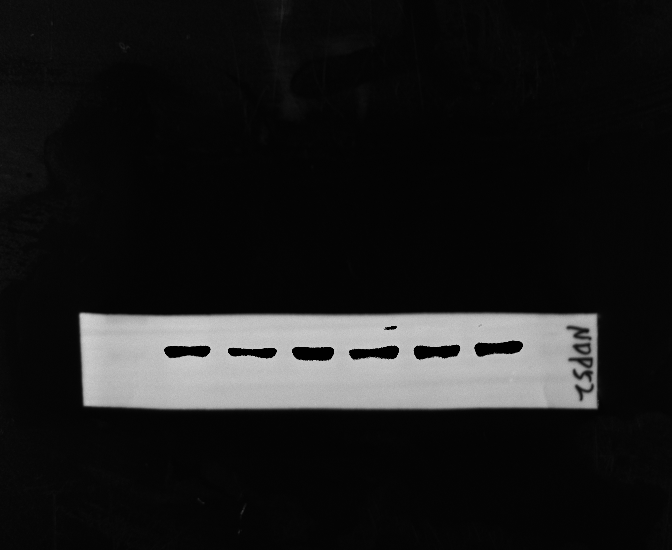

Supplement: Supplemental Information 1 [file peerj-11-16497-s001.zip › Original images for blots/original images of Human endometrial tissue/NDP52_20221031_133410_00.00.125_8bit.tif]

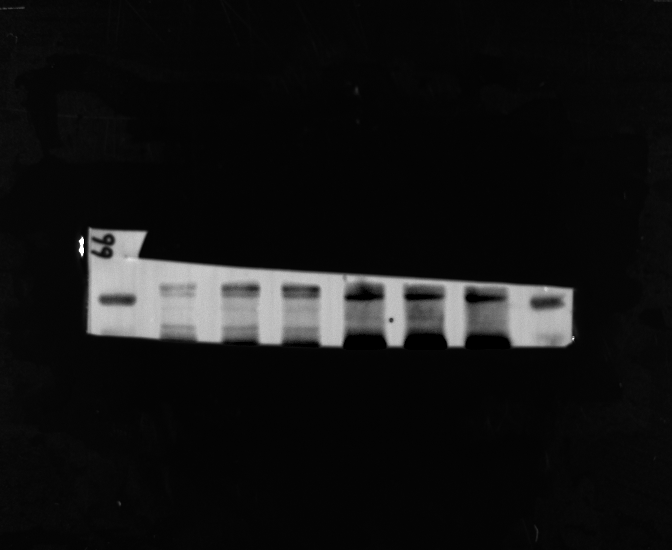

Supplement: Supplemental Information 1 [file peerj-11-16497-s001.zip › Original images for blots/original images of Human endometrial tissue/OPTN_20221031_130452_00.05.000_8bit.tif]

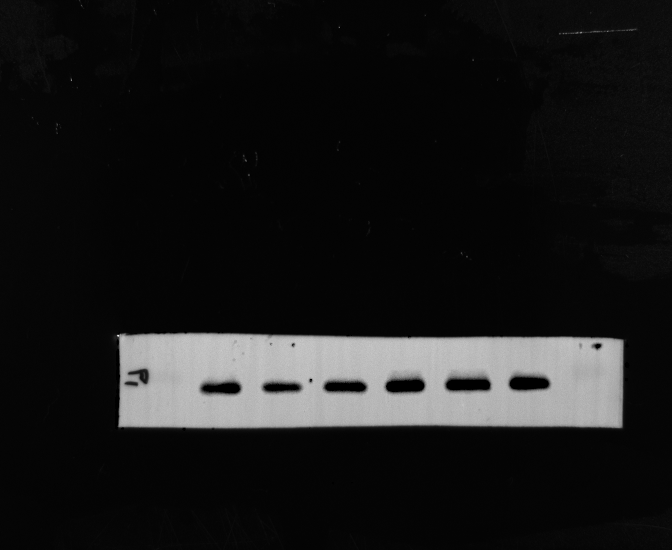

Supplement: Supplemental Information 1 [file peerj-11-16497-s001.zip › Original images for blots/original images of Human endometrial tissue/P62-1_20221105_143358_00.14.423_8bit.tif]

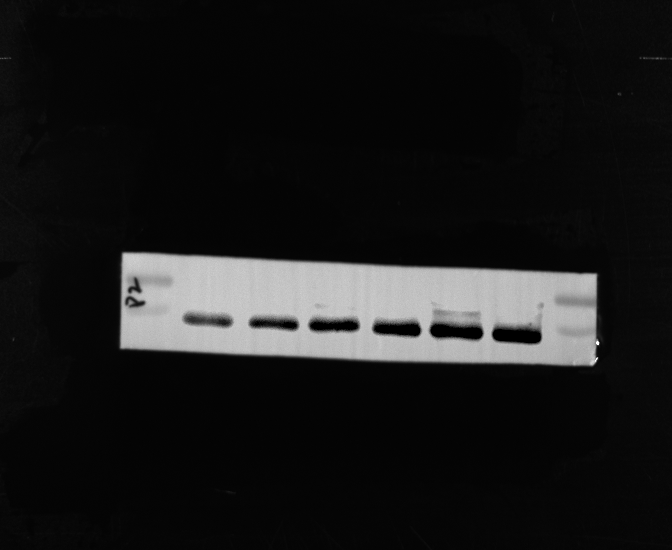

Supplement: Supplemental Information 1 [file peerj-11-16497-s001.zip › Original images for blots/original images of Human endometrial tissue/PARKIN-2_20221101_122510_00.01.480_8bit.tif]

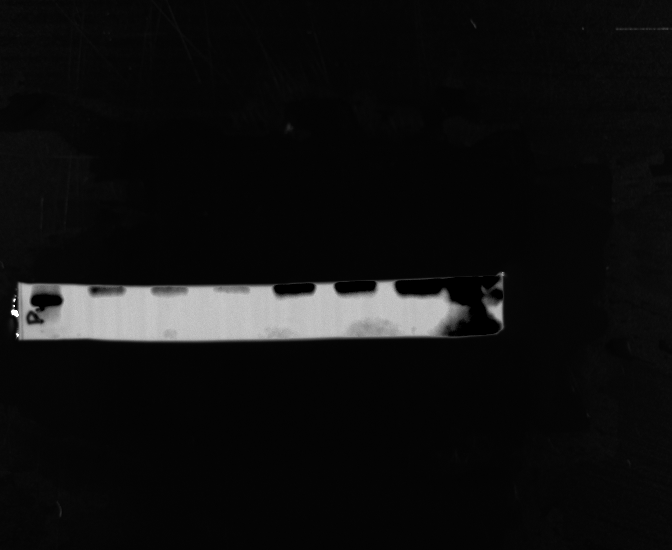

Supplement: Supplemental Information 1 [file peerj-11-16497-s001.zip › Original images for blots/original images of Human endometrial tissue/PINK1_20221031_125711_00.08.000_8bit.tif]

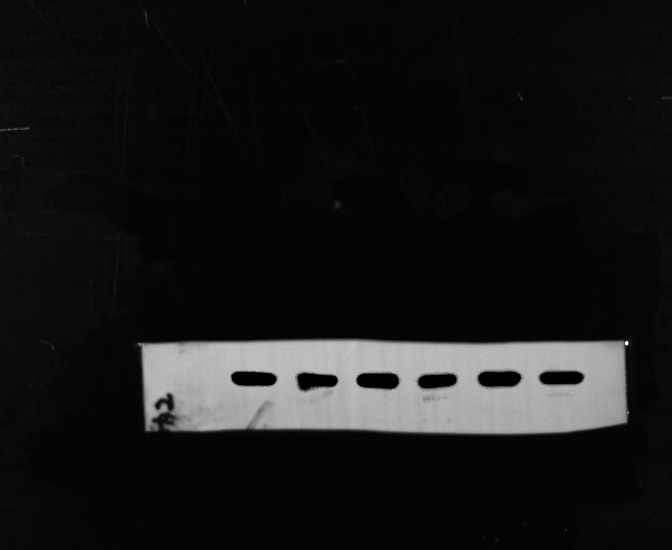

Supplement: Supplemental Information 1 [file peerj-11-16497-s001.zip › Original images for blots/original images of Human endometrial tissue/gapdh2_20221031_125306_00.00.244_8bit.tif]

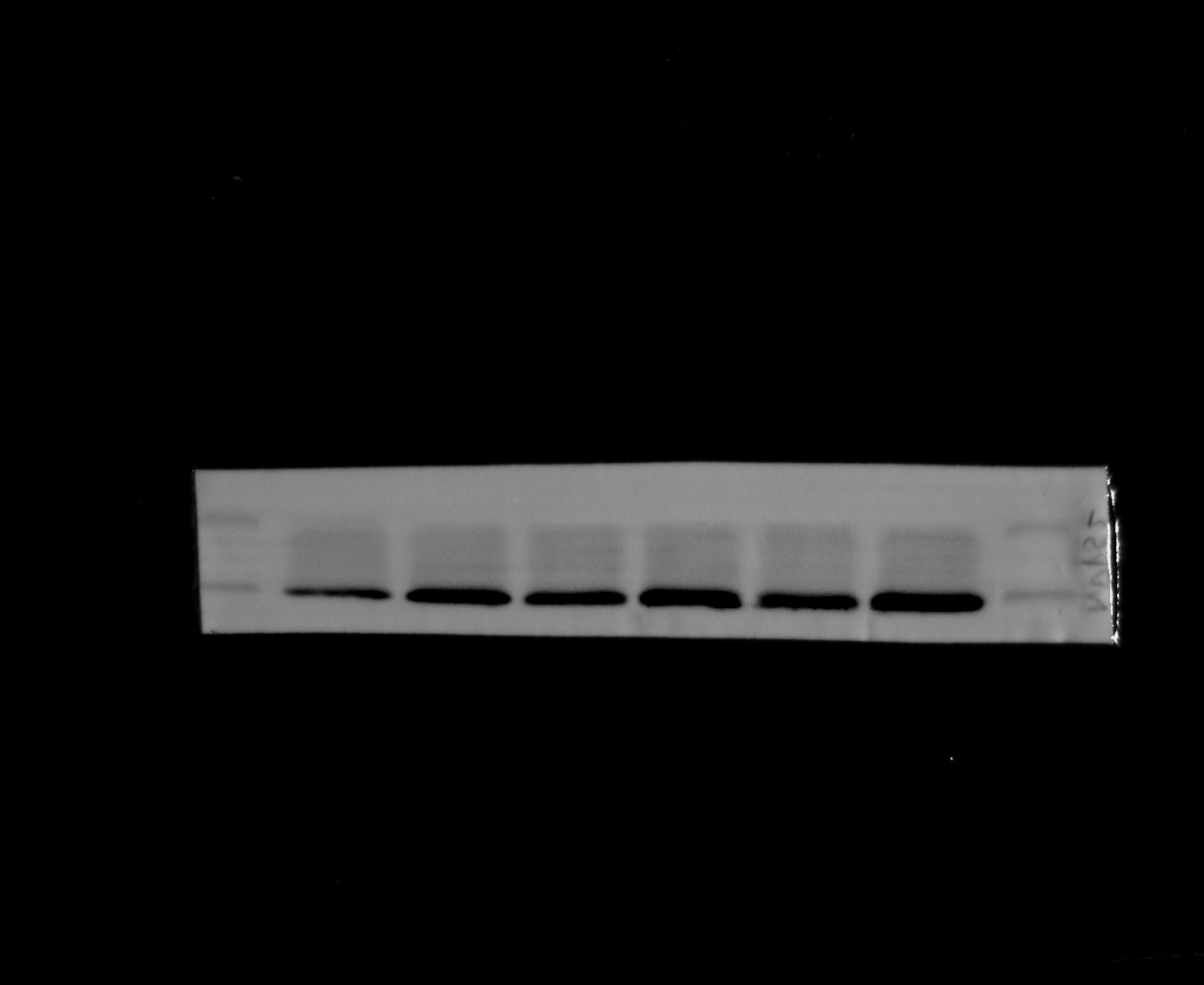

Supplement: Supplemental Information 1 [file peerj-11-16497-s001.zip › Original images for blots/original images of cells/ndp52 1_20230403_105325_00.00.194_8bit.tif]

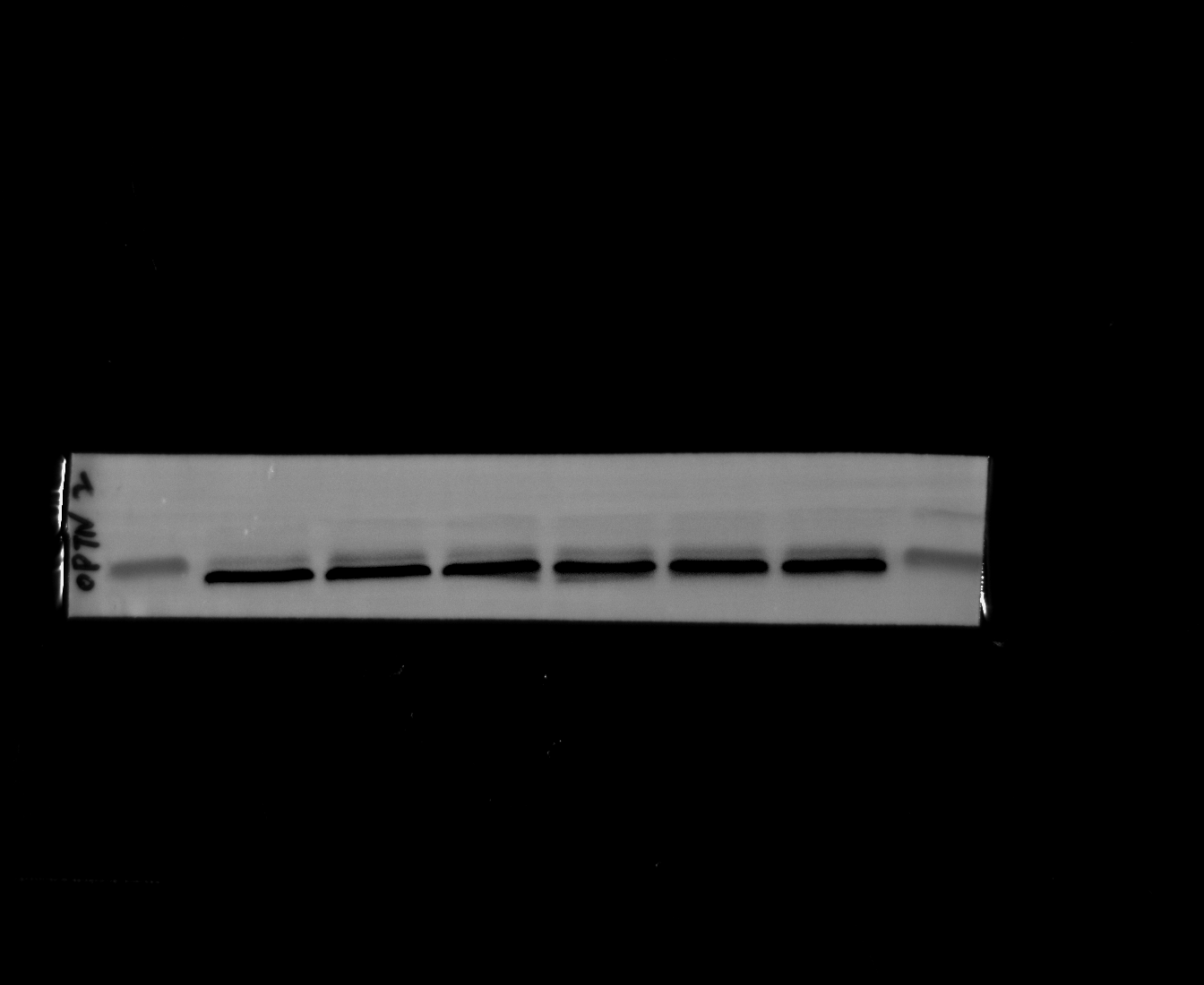

Supplement: Supplemental Information 1 [file peerj-11-16497-s001.zip › Original images for blots/original images of cells/optn_20230403_110617_00.00.245_8bit.tif]

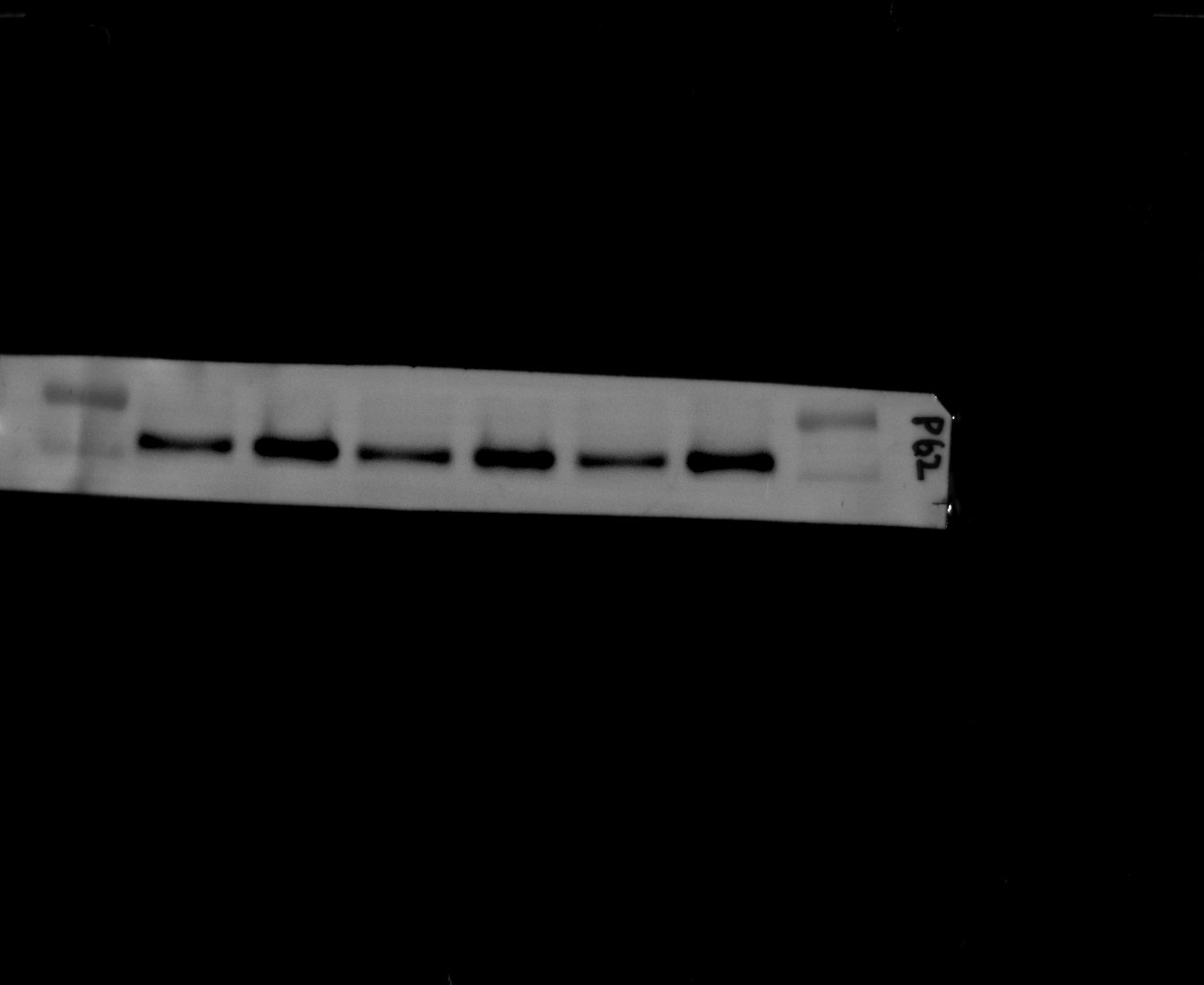

Supplement: Supplemental Information 1 [file peerj-11-16497-s001.zip › Original images for blots/original images of cells/p62_20230325_173725_00.01.805_8bit(0).tif]

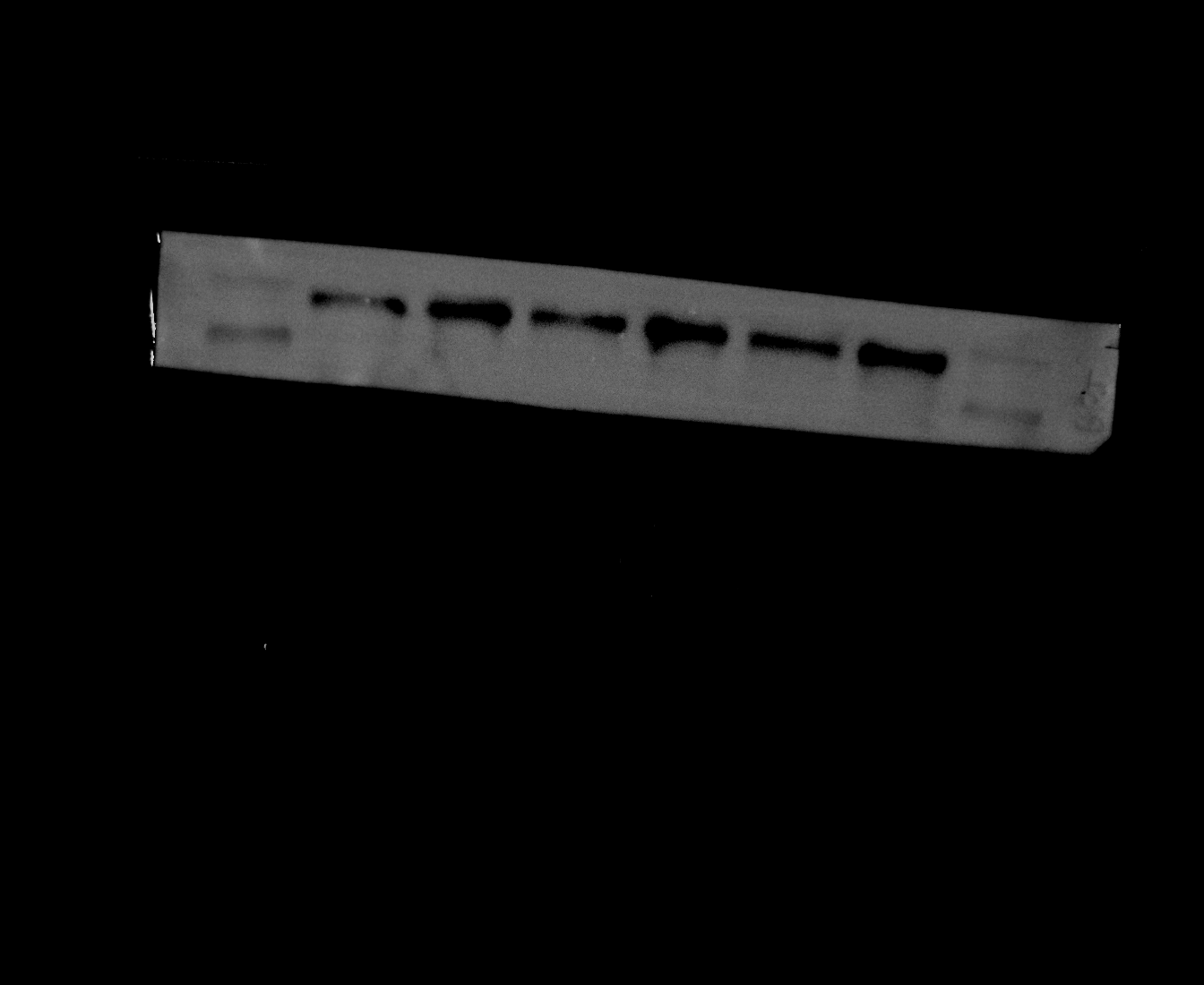

Supplement: Supplemental Information 1 [file peerj-11-16497-s001.zip › Original images for blots/original images of cells/parkin_8bit.tif]

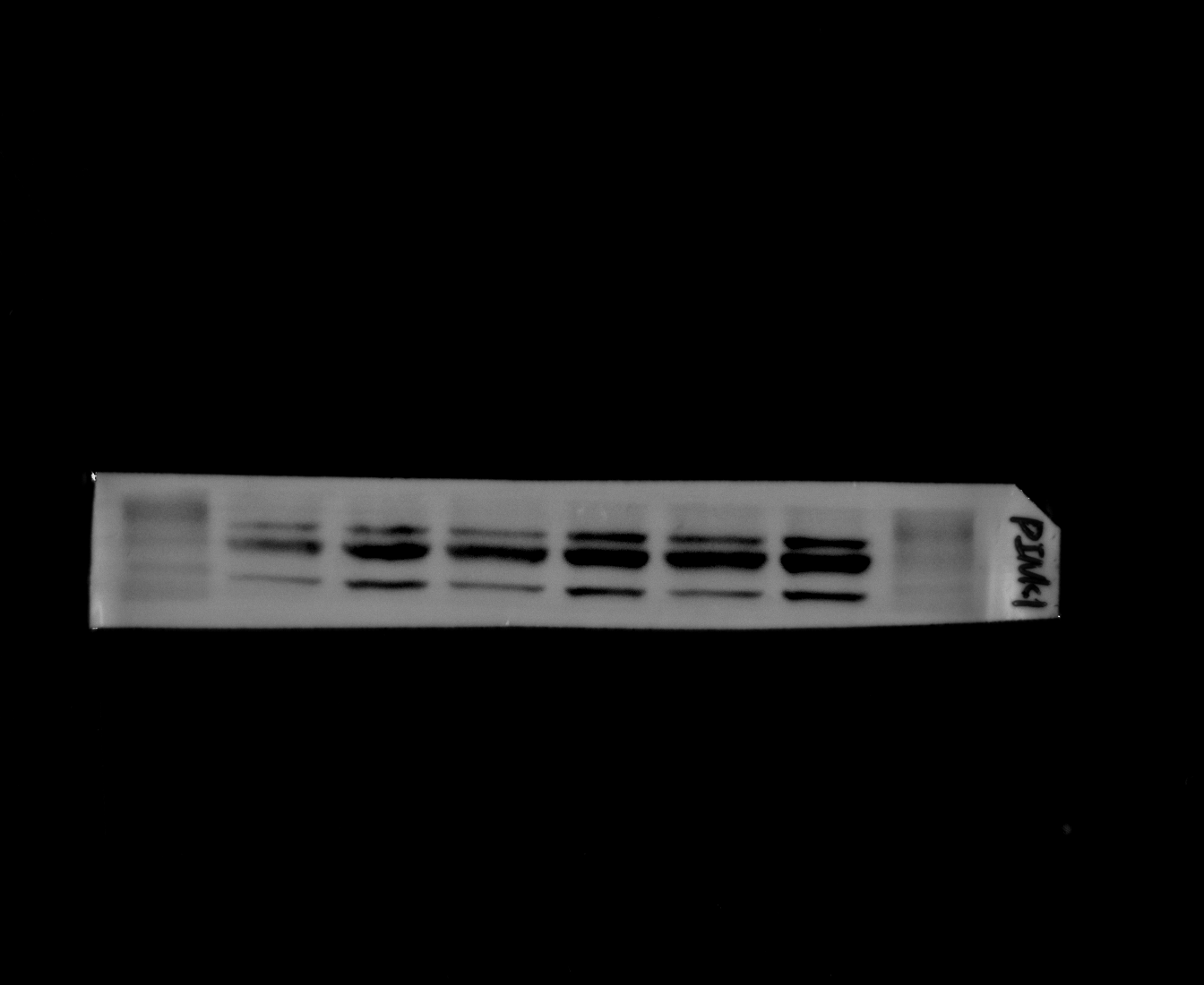

Supplement: Supplemental Information 1 [file peerj-11-16497-s001.zip › Original images for blots/original images of cells/pink1_20230325_175217_00.02.913_8bit.tif]

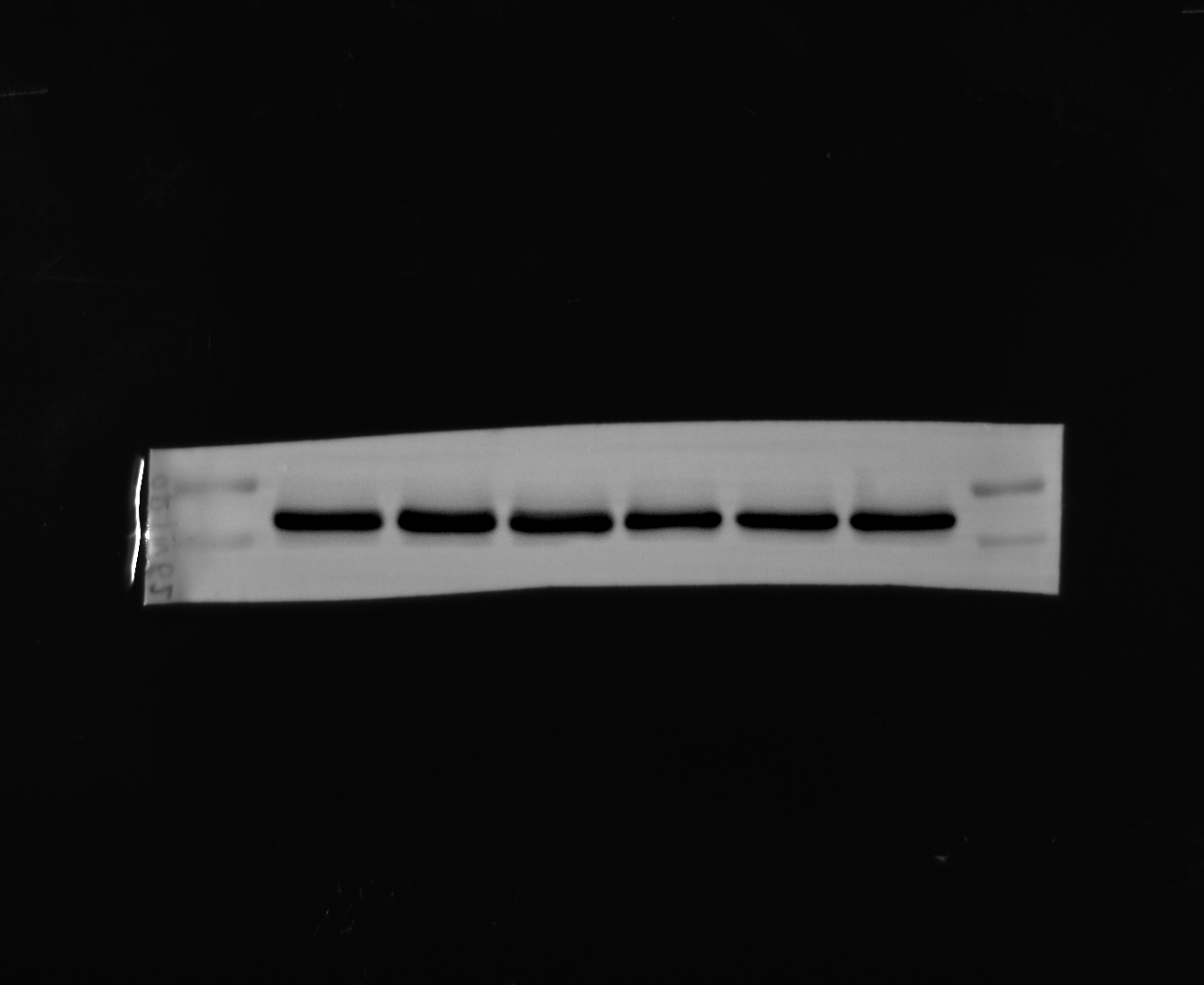

Supplement: Supplemental Information 1 [file peerj-11-16497-s001.zip › Original images for blots/original images of cells/β-actin_20230403_110104_00.00.293_8bit (2).tif]

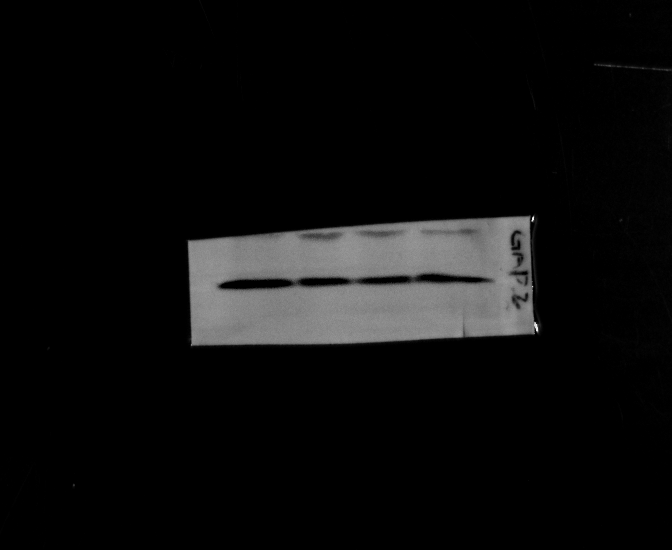

Supplement: Supplemental Information 1 [file peerj-11-16497-s001.zip › Original images for blots/original images of mice uterine tissue/GAPDH2_20230226_125657_00.00.066_8bit.tif]

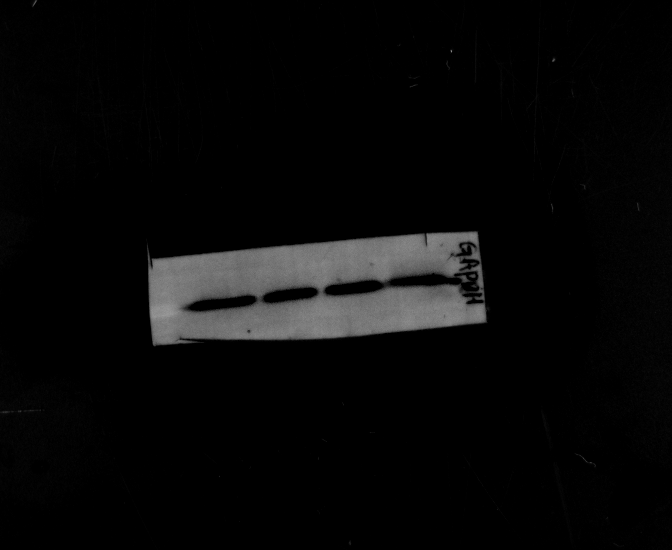

Supplement: Supplemental Information 1 [file peerj-11-16497-s001.zip › Original images for blots/original images of mice uterine tissue/GAPDH_D20230225_115557_00.00.272_8bit(0)(1).tif]

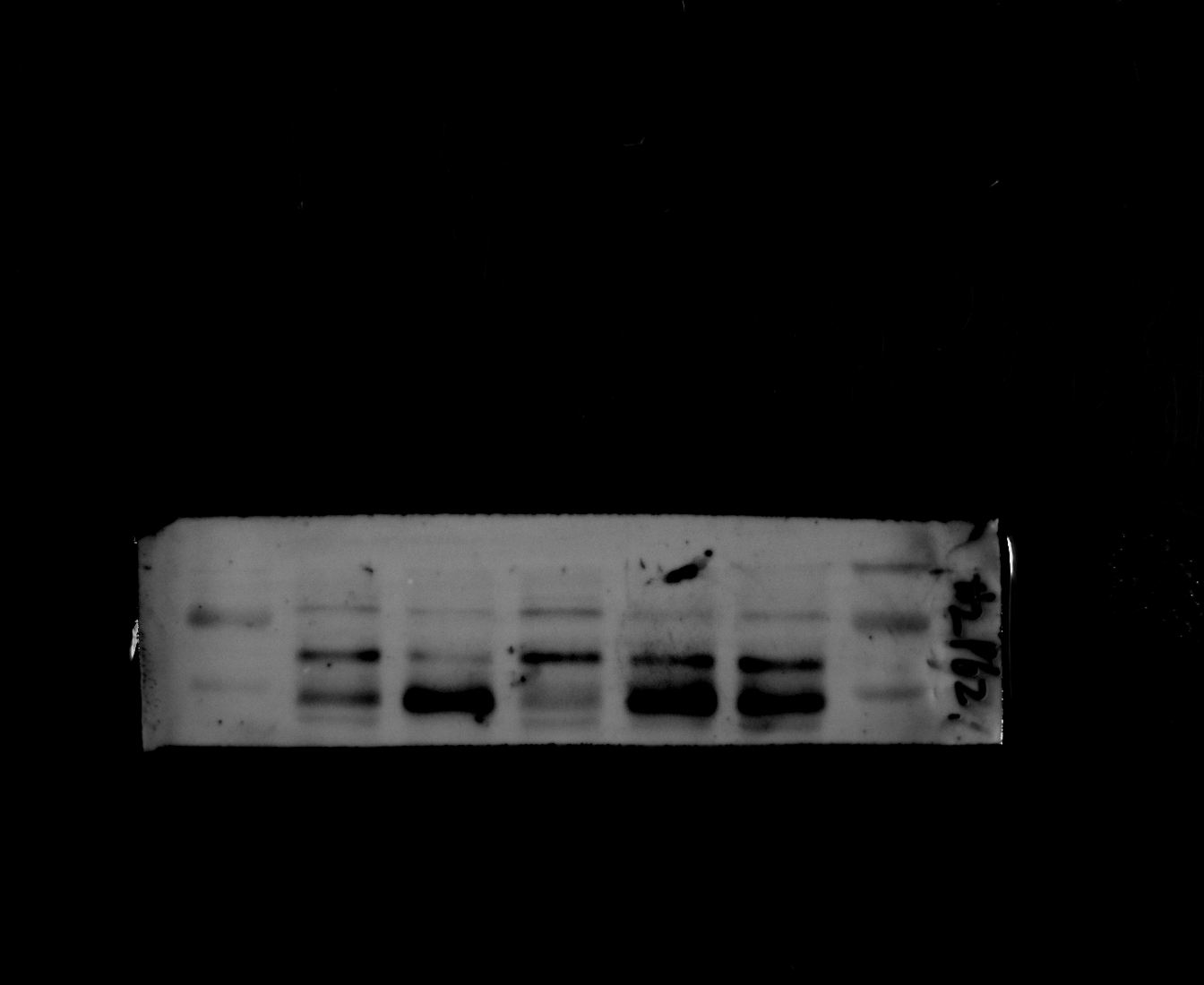

Supplement: Supplemental Information 1 [file peerj-11-16497-s001.zip › Original images for blots/original images of mice uterine tissue/P62_20230517_120909_00.32.432_8bit.tif]

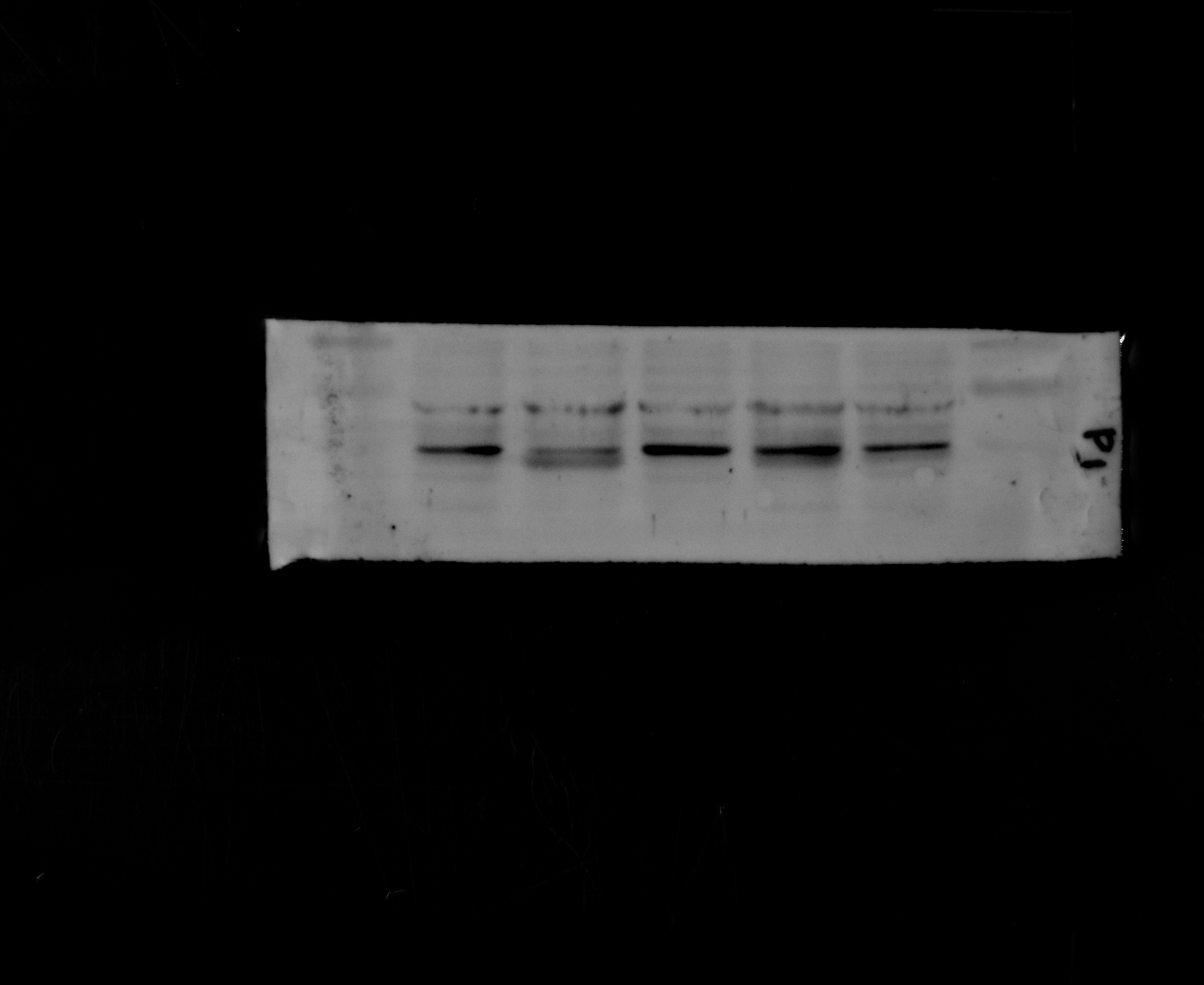

Supplement: Supplemental Information 1 [file peerj-11-16497-s001.zip › Original images for blots/original images of mice uterine tissue/PARKIN_20230517_121452_00.05.735_8bit(0).tif]

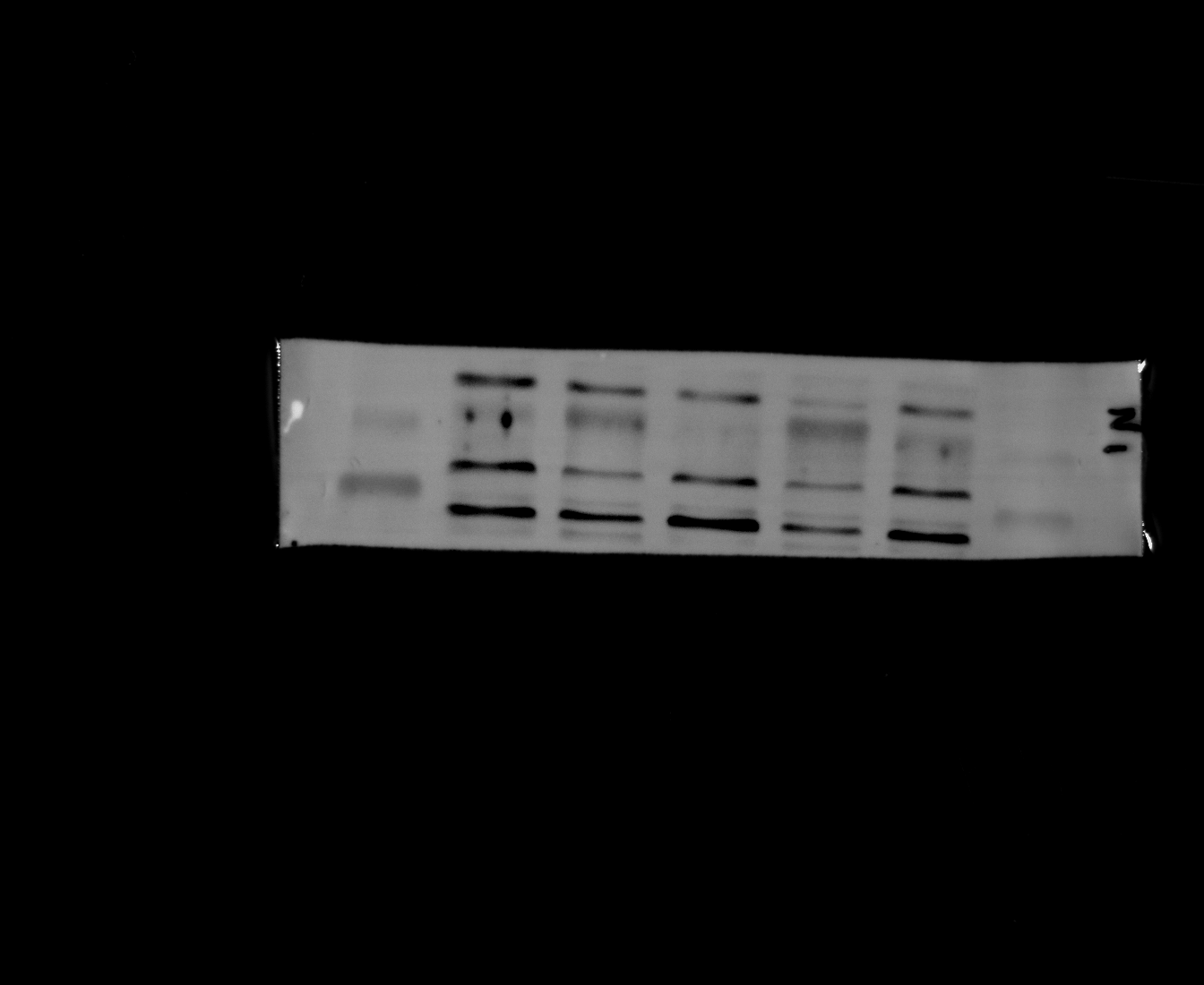

Supplement: Supplemental Information 1 [file peerj-11-16497-s001.zip › Original images for blots/original images of mice uterine tissue/ndp52_20230516_111748_00.01.815_8bit(0).tif]

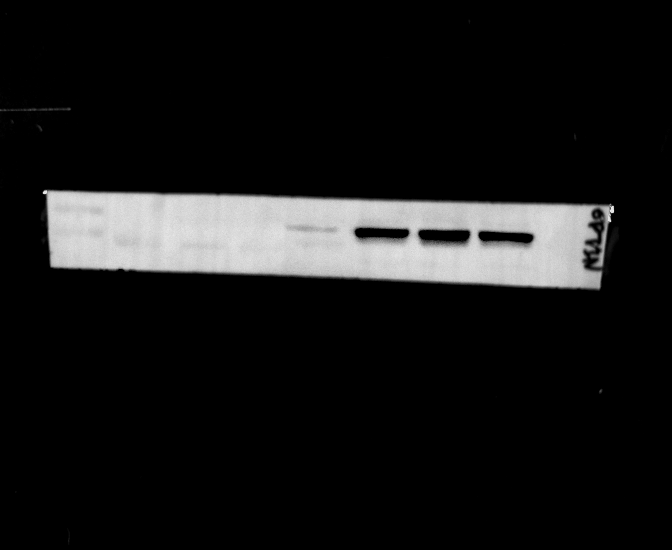

Supplement: Supplemental Information 1 [file peerj-11-16497-s001.zip › Original images for blots/original images of mice uterine tissue/optn_20230222_120155_00.01.190_8bit.tif]

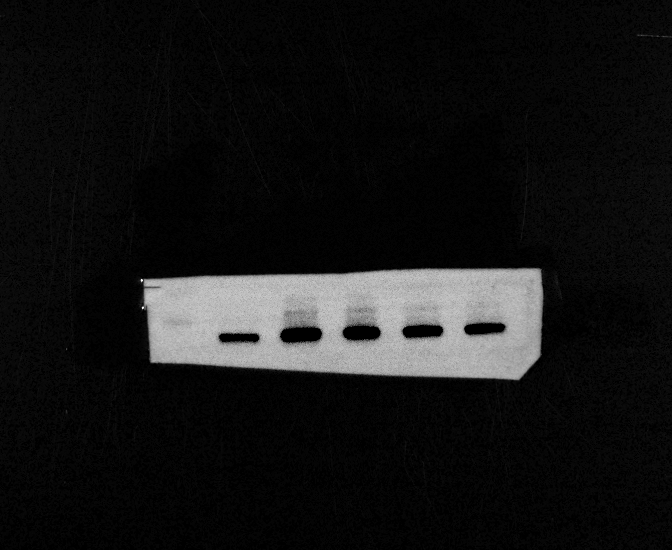

Supplement: Supplemental Information 1 [file peerj-11-16497-s001.zip › Original images for blots/original images of si-RNA/G叠加_8bit.tif]

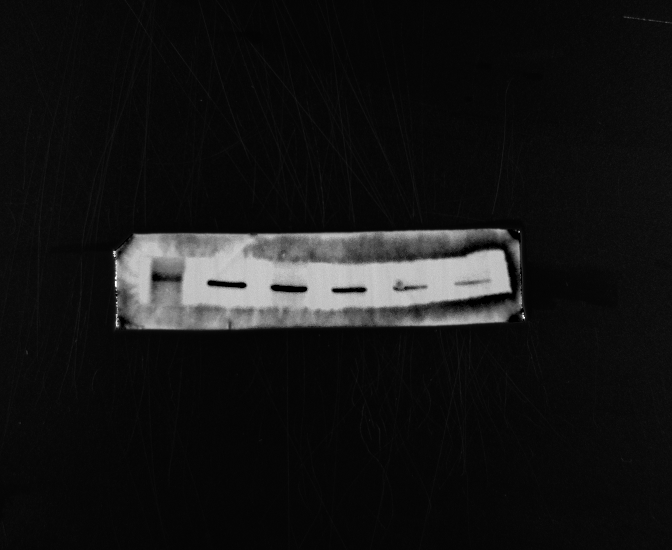

Supplement: Supplemental Information 1 [file peerj-11-16497-s001.zip › Original images for blots/original images of si-RNA/PINK1_8bit.tif]
