# Supplementary material for: Role of Pink1-mediated mitophagy in adenomyosis
Source: PeerJ. 2023 Nov 30;11:e16497. doi: 10.7717/peerj.16497 (PMC10693823; doi:10.7717/peerj.16497)
Supplement: Supplemental Information 3 [file peerj-11-16497-s003.docx]

**1 Identification of endometrial stromal cells (ESCs)**

Figure 7 illustrates the successful culture and identification process of ESCs, which involved observing a significant amount of spindle-shaped endometrial stromal cells under a inverted microscope. Through immunofluorescence staining, the cells were determined to be positive for vimentin (red), thus providing confirmatory evidence of their endometrial stromal cell origin.


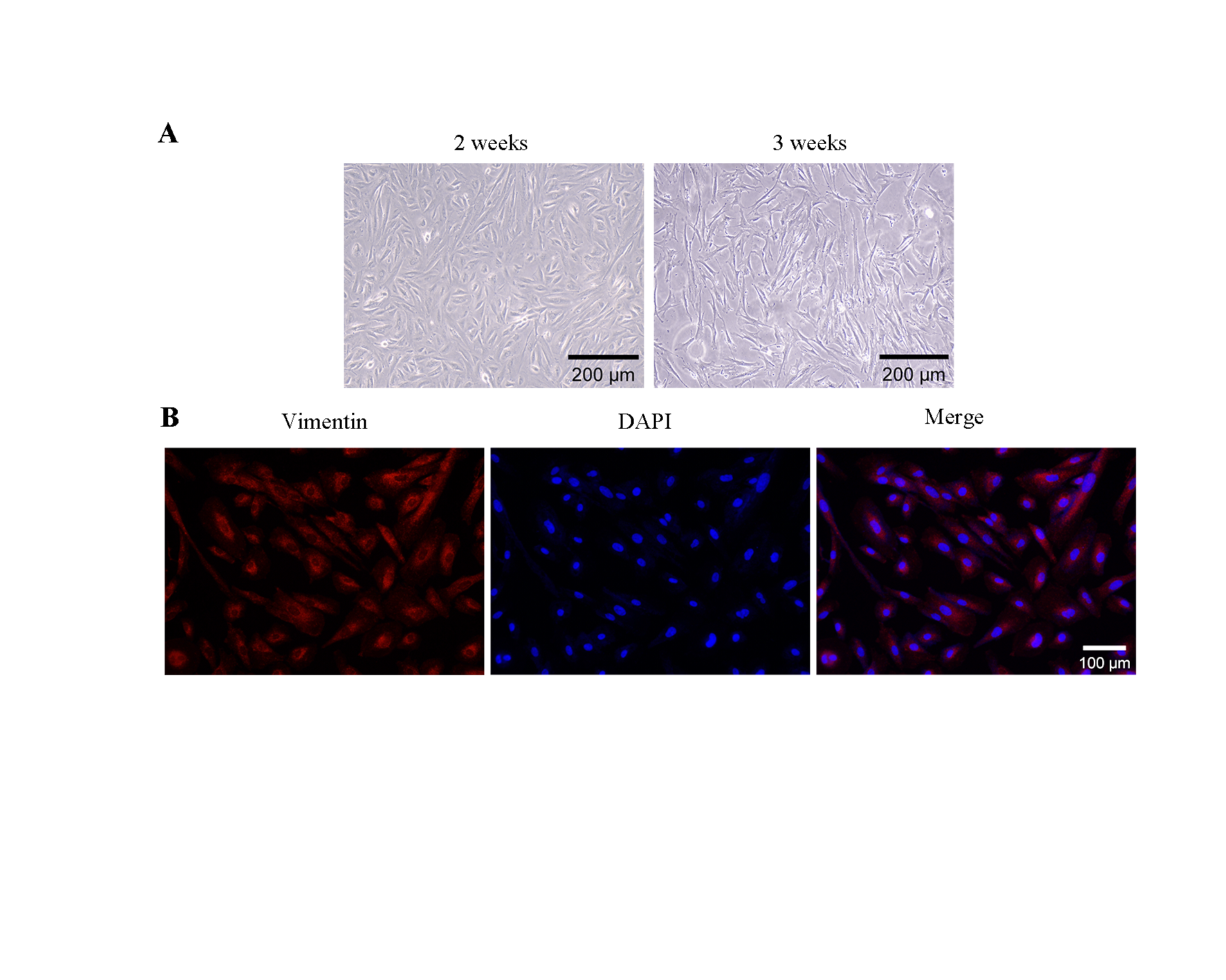


Figure 1. Culture and identification of ESC cells. A: The observation of AM-ESCs under inverted microscope; B: The mmunofluorescence staining of vimentin.

**2 Knockdown of PINK1**

Following the transfection of ESCs with siRNA for 48 hours, FAM fluorescence was observed in all groups using an inverted fluorescence microscope (Figure 10). The si-3 group exhibited the highest rate of fluorescence expression, denoting a transfection efficiency of around 80%. As a result, it can be inferred that the administrate with siRNA has effectively induced gene knockdown in ESCs.


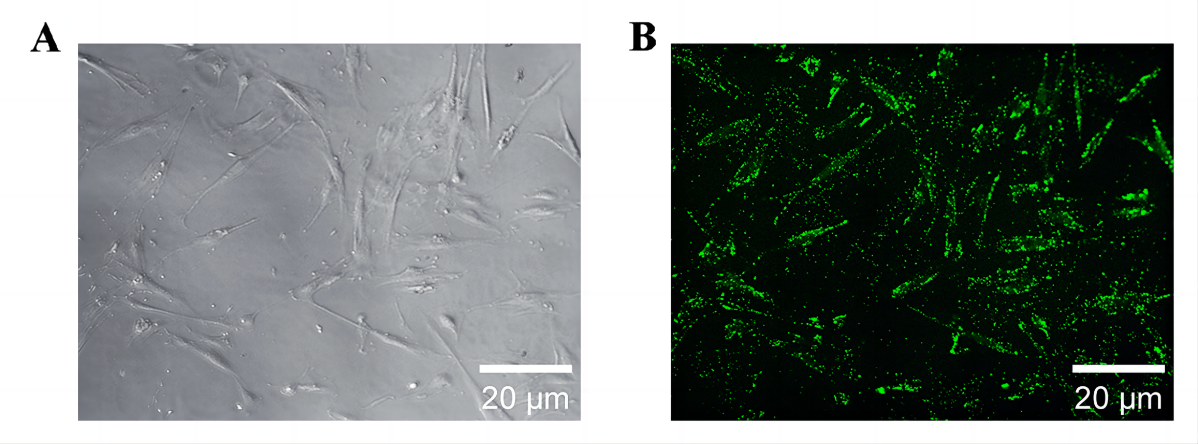


Figure 2. Transfection of siRNA with green fluorescence into ESC. A: Light microscopic observation of ESC cultured for 3 weeks; B: ESC with green fluorescence.
